# Unmanned Aerial Vehicles (UAVs) and Artificial Intelligence Revolutionizing Wildlife Monitoring and Conservation

**DOI:** 10.3390/s16010097

**Published:** 2016-01-14

**Authors:** Luis F. Gonzalez, Glen A. Montes, Eduard Puig, Sandra Johnson, Kerrie Mengersen, Kevin J. Gaston

**Affiliations:** 1 Australian Research Centre for Aerospace Automation (ARCAA), Queensland University of Technology (QUT), 2 George St, Brisbane QLD 4000, Australia; glen.montes@mail.escuelaing.edu.co (G.A.M.); eduard.puiggarcia@qut.edu.au (E.P.); 2 ARC Centre of Excellence for Mathematical & Statistical Frontiers (ACEMS), Queensland University of Technology (QUT), 2 George St, Brisbane QLD 4000, Australia; sandra.johnson@qut.edu.au (S.J.); k.mengersen@qut.edu.au (K.M.); 3 Environment and Sustainability Institute, University of Exeter, Penryn, Cornwall TR10 9EZ, UK; k.j.gaston@exeter.ac.uk

**Keywords:** Unmanned Aerial Vehicle (UAV), wildlife monitoring, artificial intelligence, thermal imaging, robotics, conservation, automatic classification, koala, deer, wild pigs, dingo, conservation

## Abstract

Surveying threatened and invasive species to obtain accurate population estimates is an important but challenging task that requires a considerable investment in time and resources. Estimates using existing ground-based monitoring techniques, such as camera traps and surveys performed on foot, are known to be resource intensive, potentially inaccurate and imprecise, and difficult to validate. Recent developments in unmanned aerial vehicles (UAV), artificial intelligence and miniaturized thermal imaging systems represent a new opportunity for wildlife experts to inexpensively survey relatively large areas. The system presented in this paper includes thermal image acquisition as well as a video processing pipeline to perform object detection, classification and tracking of wildlife in forest or open areas. The system is tested on thermal video data from ground based and test flight footage, and is found to be able to detect all the target wildlife located in the surveyed area. The system is flexible in that the user can readily define the types of objects to classify and the object characteristics that should be considered during classification.

## 1. Introduction

Effective management of populations of threatened and invasive species relies on accurate population estimates [1]. Existing monitoring protocols employing techniques such as remote photography, camera traps, tagging, GPS collaring, scat detection dogs and DNA sampling typically require considerable investment in time and resources [2,3]. Moreover, many of these techniques are limited in their ability to provide accurate and precise population estimates [4]. Some of the challenges in wildlife monitoring include the large size of species’ geographic ranges [5], low population densities [3], inaccessible habitat [6,7], elusive behaviour [8] and sensitivity to disturbance [9]. 

The increase in availability of inexpensive Unmanned Aerial Vehicles (UAVs) provides an opportunity for wildlife experts to use an aerial sensor platform to monitor wildlife and tackle many of these challenges to accurately estimate species abundance [10,11,12]. In recent years, the use of UAVs that can perform flight paths autonomously and acquire geo-referenced sensor data has increased sharply for agricultural, environmental and wildlife monitoring applications [13,14,15]. Some issues restricting the wider use of UAVs for wildlife management and research include UAV regulations [9,15,16,17], operational costs and public perception. One of the most important restrictions, however, is the need to develop or apply advanced automated image detection algorithms designed for this task. 

Current examples of the use of UAVs for wildlife management include monitoring sea turtles [18], black bears [8], large land mammals (e.g., elephants [19]), marine mammals (e.g., dugongs [20]) and birds (e.g., flocks of snow geese [21]), wildlife radio collar tracking [22], and supporting anti-poaching operations for rhinos [23]. UAVs with digital and thermal imagery sensors can record high resolution videos and capture images much closer to the animals than manned aerial surveys with fewer disturbances [9,10,18,22]. Jones *et al.* [24] for example conducted a test that involved gathering wildlife video and imagery data from more than 30 missions over two years, and concluded that a UAV could overcome “safety, cost, statistical integrity and logistics” issues associated with manned aircraft for wildlife monitoring. Other advances in this field include autonomous tracking of radio-tagged wildlife [13,25,26]. 

Overall, UAVs have proven to be effective at carrying out wildlife monitoring surveys however in many cases, the extensive post-processing effort required negates any convenience or time savings afforded by UAVs in the field compared to conventional survey methods. Therefore, for UAVs to become truly efficient wildlife monitoring tools across the entire workflow of data collection through to analysis, improved capabilities to automate animal detection and counting in the imagery collected by UAVs are required. Research into automatic classification of UAV images for wildlife monitoring is emerging. For example, van Gemert *et al*. [14] evaluated the use of UAVs and state-of-the-art automatic object detection techniques for animal detection demonstrating a promising solution for conservation tasks. Although using an elevated structure rather than a UAV, Christiansen *et al.* [4] used thermal imagery and a k-nearest-neighbour classifier to discriminate between animal and non-animal objects, achieving 93.3% accuracy in an altitude range of 3–10m. In this paper, we further address the issue of automated wildlife detection in UAV imagery by describing a system composed of a UAV equipped with thermal image acquisition as well as a video processing pipeline to perform automated detection, classification and tracking of wildlife in a forest setting to obtain a population estimate within the area surveyed.

## 2. Experimental Design

### 2.1. System Architecture

**Figure 1:**
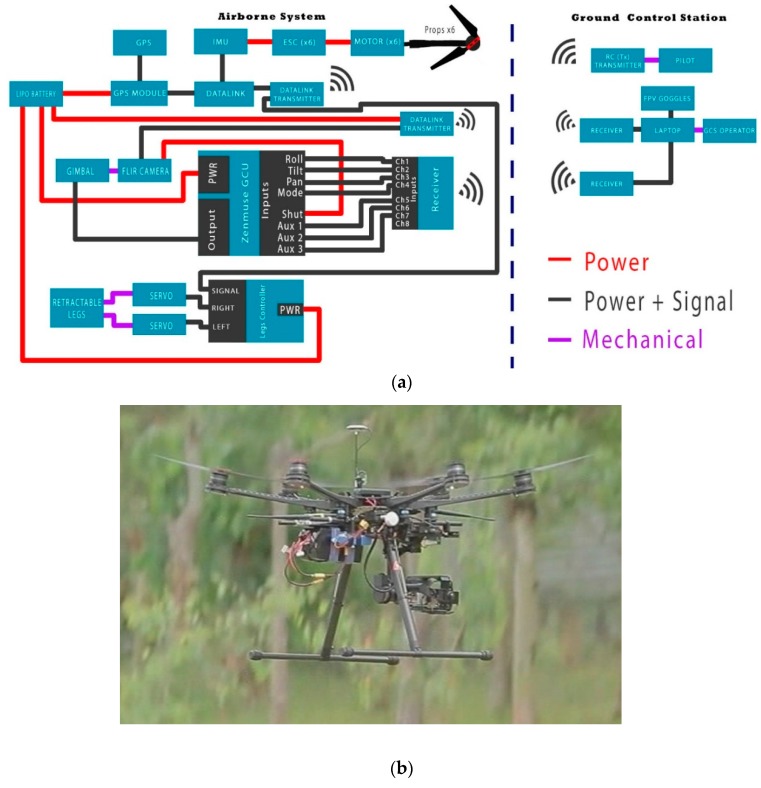
(**a**) System Architecture consisting of airborne and ground control segments and (**b**) Multirotor UAV, thermal camera, gimbal system and video transmitter.

The system used in this experiment can be divided into airborne and ground segments as presented in Figure 1a. The airborne system consists of the multirotor UAV, navigation system, thermal camera, gimbal system and video transmitter (Figure 1b). The ground segment consists of the ground station software installed in a laptop, the datalink and video receivers for remote display and recording. 

#### 2.1.1. Unmanned Aerial Vehicle (UAV)

The aerial platform weighs approximately 6 kg, including flight and communications systems. It has a recommended maximum take-off weight of 8 kg, thus allowing 2 kg for sensor payload. The UAV has four main sub-systems: the airframe, the power and propulsion subsystem, the navigation subsystem and the communications subsystem. These subsystems are integrated to provide navigation and power during the UAV flight operations.

##### Airframe

The airframe used in this platform is an S800 EVO Hexacopter [27] weighing 5.4 kg with motors, propellers and ESCs (electronic speed controllers). The frame is fitted with a retractable undercarriage, providing a sensor field of view clear of obstacles. 

##### Power and Propulsion

The UAV uses a 16,000 mAh Lipo 6 cell battery. This provides a maximum hover time of approximately 20 mins with no sensor payload. The maximum motor power consumption of each motor is 500 W operating at 400 rpm/V. These are running in conjunction with 15 × 5.2 inch propellers.

##### Navigation

The main component of the navigation system is a WooKong-M (WK-M) flight controller autopilot, which comes with a GPS unit with inbuilt compass, stabilization controller, gimbal stabilizer, position and altitude hold, auto go home/landing, with enhanced fail-safe. The system has an IMU located in the centre of the UAV to reduce the vibrations and reduce the risk of damage or failure. The autopilot’s role in the aircraft is to navigate towards the desired location by altering the altitude, direction and speed. The autopilot has three main operating modes. The first mode is autonomous which allows the UAV to fly a predefined flight path that is designed using the ground control station (GCS). The second mode is stabilized mode which is designed for pre-flight checks of the control surfaces and autopilot. This mode allows the aircraft to maintain a level flight when no pilot input is received. The final mode is full manual which is generally used for take-off and landings, as well as any emergency situations. The GPS connects directly to the autopilot multi-rotor controller as seen in Figure 1.

##### FLIR Camera, Gimbal System and Video Transmission

**Figure 2:**
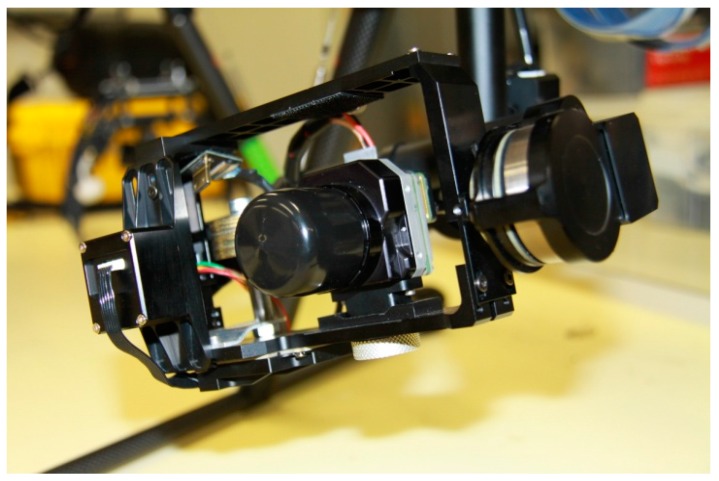
FLIR camera attached to the gimbal system.

The FLIR camera used is a Tau 2-640 [28], Figure 2. The camera weighs 100 g and has a 640 × 480 pixels resolution and 25 mm focal lens. The FLIR has a field of view of 25 × 22 degrees. The FLIR video can be sent to the ground via AVL58 5.8 GHz Video Link which comprises a receiver in the laptop and an airborne transmitter. The video received by the laptop is recorded with off-the-shelf video recording software. The sampling frequency of the thermal camera is 9 fps and the sensitivity (NEdT) is <50 mK at f/1.0. The scene range (high gain) is −25° to +135° and the low gain is −40° to +550°. The camera is capable of operating at altitudes up to +40,000 ft and has an operating temperature of −40 °C to +80 °C [28]. A gimbal system is used to hold the camera as shown in Figure 2, stabilize the video signal and dampen the vibrations of the frame. The gimbal control commands are received by the receiver attached to the Zenmuse Gimbal Control Unit (GCU). This allows the pilot or UAV controller to change the roll, pitch, pan and mode of the gimbal, and to control the FLIR camera.

##### Ground Control Station and Datalink

The ground station software together with the datalink enables flight path planning with predefined waypoints and real-time telemetry reception in the ground station. The datalink communication between the UAV and the ground station is performed with a LK900 Datalink that consists of an airborne transmitter and a receiver connected to the ground station laptop. With a frequency of 900 MHz, the range of the datalink is estimated to be up to 1 km outdoors in optimal conditions. This hardware and software combination ensures stable flight performance while providing real-time flight information to the UAV operations crew. Although it is possible to take-off and land autonomously, it is recommended that these flight segments are completed manually via radio transmitter to guarantee safety standards.

##### Remote Display for Visualization

**Figure 3:**
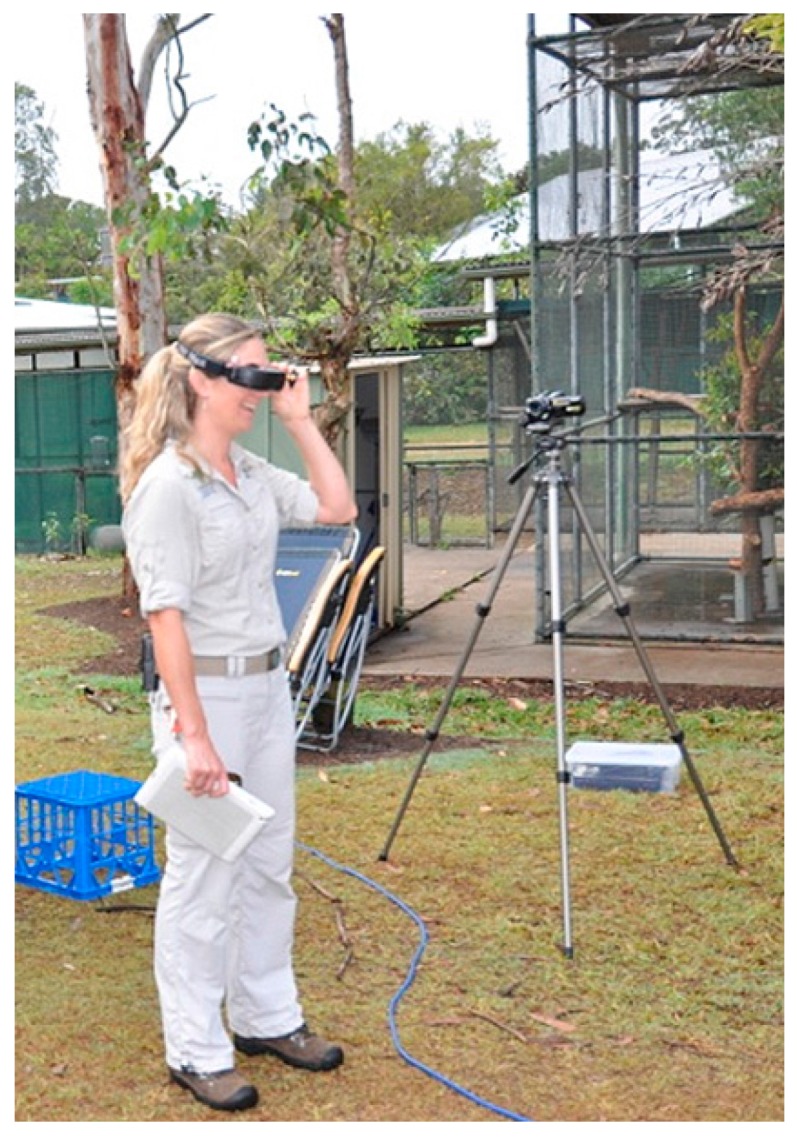
Wildlife expert using FPV goggles to observe wildlife while the UAV is flying above the canopy.

The ground station is equipped with a First Person View (FPV) system, to visualize the thermal video in real-time. The FPV goggles (Fatshark Dominator) may be used by UAV crew members, ecologists and wildlife experts to monitor aircraft motion in relation to wildlife as the UAV flies over the tree canopy (Figure 3).

### 2.2. Algorithms for Counting and Tracking

We implemented two algorithms on the ground control station computer that automatically count, track and classify wildlife using a range of characteristics. Different approaches are required depending on the information provided by the image or video. For instance, using a clear image of a koala, deer or a kangaroo, colour, size and position thresholds are applied to determine the object of interest. More complex algorithms are required if the image is less clear, for example if the object is an irregular shape, with no apparent colour, of variable size or in multiple positions. The algorithms were written in the Python programming language using the SimpleCV framework for ease of access to open source computer vision libraries such as OpenCV. 

#### 2.2.1. Algorithm 1: Pixel Intensity Threshold (PIT)

**Figure 4:**
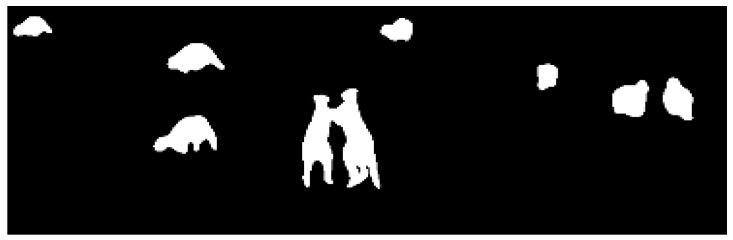
Image binarization in algorithm 1.

This algorithm approaches the problem by using the wildlife’s heat signature which creates a good contrast between the background and the target wildlife. This contrast enables an intensity threshold to be applied which in turns eliminates the background and brings the object of interest to the front. Intensity threshold, also known as binarization or segmentation of an image, assigns 0 to all pixels under or equal to the threshold and 255 to all the pixels above the same threshold where 0 represents the black colour and 255 represents the white colour (Figure 4).

The algorithm uses the following function:

p = image(x,y)

$$f\left(p\right)=\{\begin{array}{c} \;p\leq T,0\\ \;p>T,\;255\; \end{array}$$

where x and y are the coordinates of a pixel within the image and f (p) is a function that changes in value with respect to the threshold T. 

**Figure 5:**
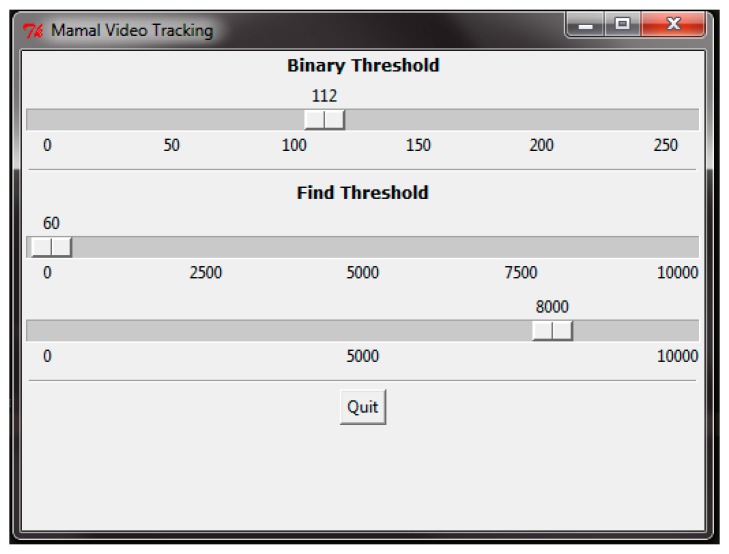
Graphical user interface (GUI) of the intensity threshold algorithm which allows the end user to adjust the thresholds using the sliders.

**Figure 6:**
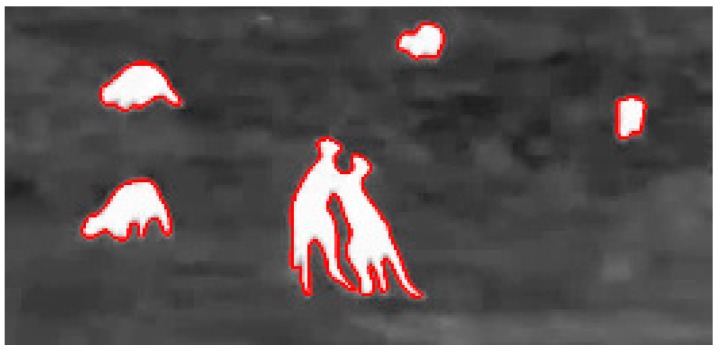
Two kangaroos appear to be one object.

A Graphical User Interface (GUI) was implemented to change this threshold using Tkinter libraries and to assist in finding the most appropriate value for T (Figure 5, slide 1). The second step is to add a morphological operation. Frequently, after applying the intensity threshold, two or more objects may appear as one because they are close to each other, causing a miscount of the number of objects in the frame (Figure 6).

The morphological operations comprise erosion and dilation steps that clean and separate objects. This process does not differentiate two objects that are on top of one another.

**Figure 7:**
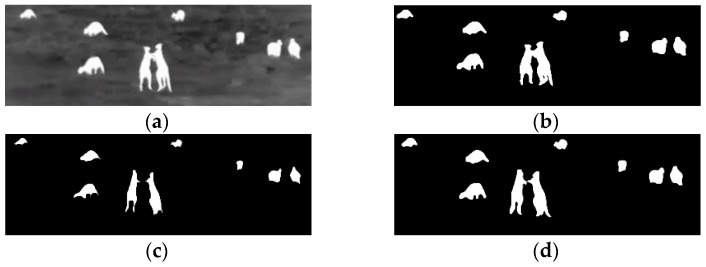
Output from PIT algorithm: (**a**) Original footage (**b**) Image binarized (**c**) Erode operation result (**d**) Dilation operation result.

As seen in Figure 7, after applying first the erosion operation and then the dilation, the PIT algorithm is able to separate the kangaroos into two different objects. The third step is to search for the object of interest in the resulting image. Defining a minimum and maximum size threshold (Figure 5, slide 2 and 3) the algorithm searches for clusters of white pixels within the range and groups them to then display and count the clusters. The minimum and maximum size is a function of the number of pixels in the objects of interest. Every threshold value described in this algorithm may be changed during video processing to accommodate changing external conditions such as light, environmental temperature and video quality. The GUI provides slides to adjust the values and fine-tune the final processing. 

**Figure 8:**
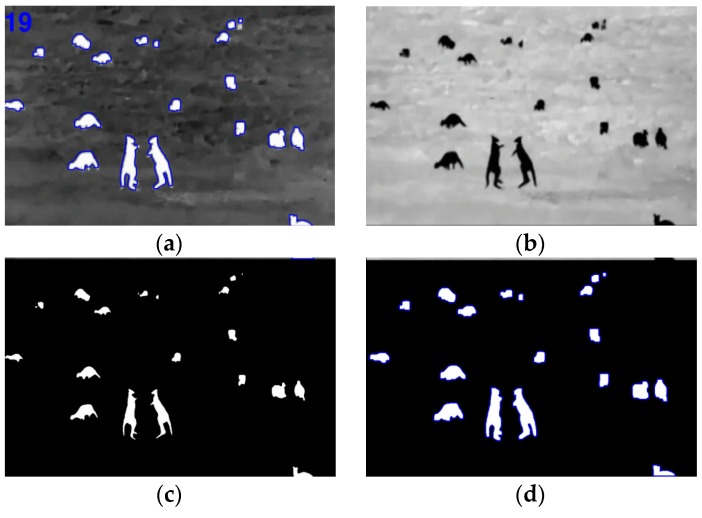
(**a**) Result footage for kangaroos (**b**) Image inverted (**c**) Erode operation (**d**) Dilation and group.

**Figure 9:**
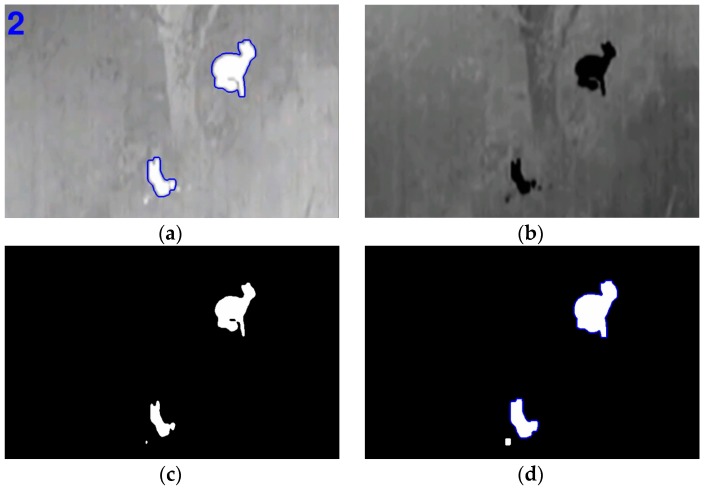
(**a**) Result footage for deer (**b**) Image inverted (**c**) Erode operation (**d**) Dilation and group.

The complete process can be seen in Figure 8 and Figure 9 for kangaroo footage [29] and deer footage [30], respectively.

Even though the PIT algorithm works well in many cases, it is dependent on good quality video and good background contrast. Some limitations of the algorithm are the inability to distinguish or classify different species and to track a single object. In order to address these limitations and to improve the processing, a second algorithm was developed.

#### 2.2.2. Algorithm 2: Template Matching Binary Mask (TMBM)

Using a reference image template of the target object, the TMBM algorithm searches for a match in each frame of the video and labels it. The template reflects characteristics of the object of interest. Multiple templates that reflect changes in size, shape and colour of the object can increase the probability of finding the object in the image. The template matching provides the starting point to detect the species of interest. Very clear and distinct differences from the thermal heat signature and the environment at close range are then used to improve the detection. 

**Figure 10:**
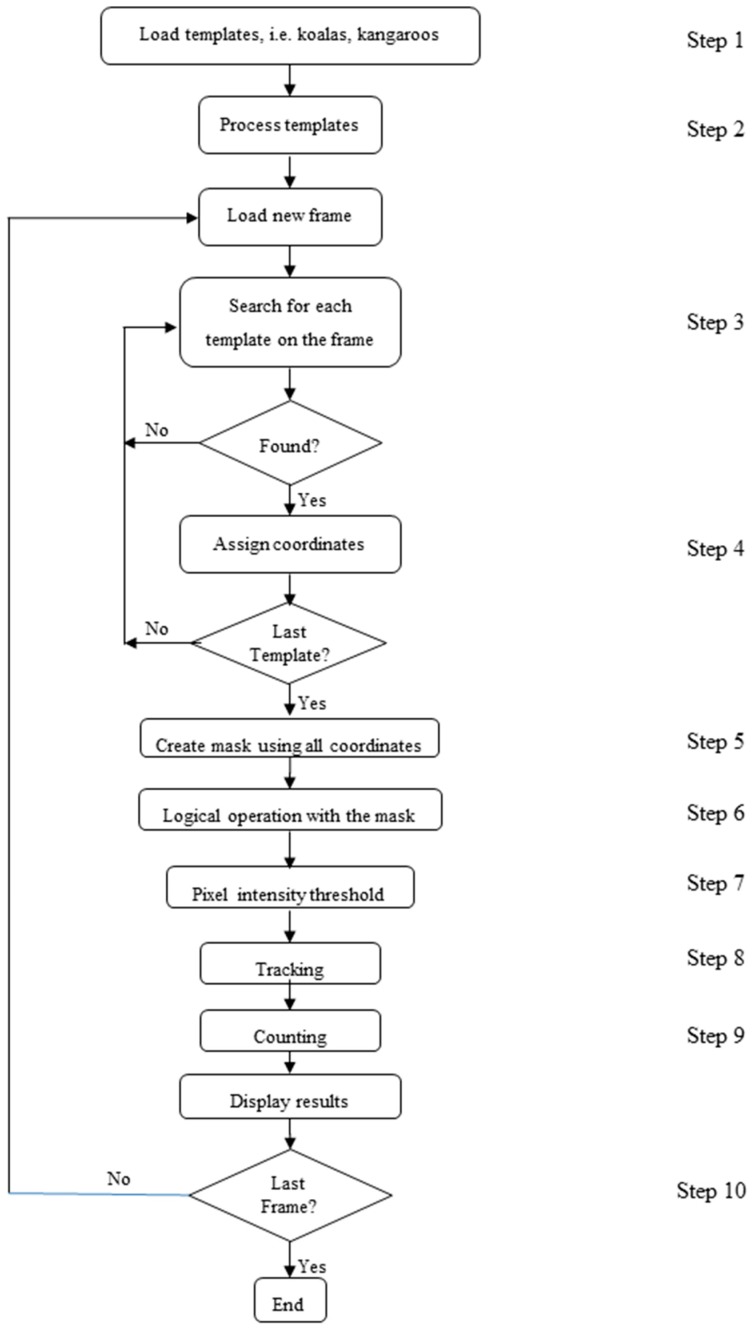
Flowchart Template Matching Binary Mask Algorithm.

**Figure 11:**
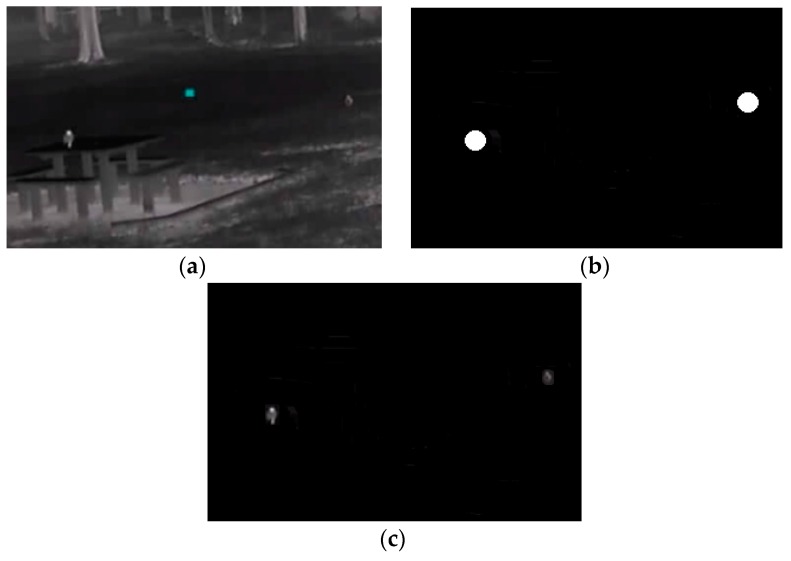
(**a**) Original footage (**b**) Mask created using the target’s coordinates (birds) (**c**) Image result eliminating the background.

The algorithm shown in Figure 10 consists of 10 main steps, as follows:

1. *Load templates:* In this first step template images are selected (e.g., koala, kangaroo, deer, pigs or birds). The templates are taken from the original footage or a database of images and then saved as small images of the object of interest. The algorithm is able to search for multiple templates in each frame.
2. *Processes Templates:* The contrast of the template is increased in order to enhance the possibility of finding this template in the footage; white is made lighter and black darker by adding or subtracting a constant, C, from each pixel value, p, depending on a threshold, T.
  p = image(x,y)
  $$f\left(p\right)=\{\begin{array}{c} p+C,\;p>T\\ p-C,\;p\leq T \end{array}$$
  The values of T and C may be changed in the program code, depending on the template quality and animal size, and can be determined by experimenting with different cases.
3. *Search for each template in the video frame:* For a detected animal to be recorded as a match it must be able to pass a scoring threshold. The searching function returns a score from 1 to 10, based on the proximity of the template to the matched object, where 1 indicates the smallest chance of finding the target and 10 indicates a perfect match. A score of 7 was designed to reflect a high quality match that gave an acceptable chance of avoiding false positives. In addition, to avoid false positives, any match found has to be present for at least 10 consecutive frames before it is considered to be a true match.
4. *Assign coordinates:* once a match has been found, the pixel coordinates (x, y) of the location within the frame are stored for later use.
5. *Create a mask using coordinates:* A new image is created with the same dimension as the source footage with a black (pixel with a value of 0) background. Using the coordinates within the image of the match found in the previous step, a white (pixel with a value of 255) bounding box or circle is drawn with a variable area (Figure 11b). The size of this area is defined by calculating the area of the match. The mask image aims to reduce the search area, eliminating what is considered as background.
6. *Logical operation with the mask:* In this step a logical AND is applied using the current frame and the mask. As a result the background is eliminated leaving only the regions of interest at the front.
7. *Pixel intensity threshold:* In this step the function described in Step 2 is used to assign a 0 if the pixel value is less than or equal to the threshold and 255 otherwise.
8. *Tracking:* After obtaining an image comprising only the objects, a function to track is implemented. This function is capable of identifying multiple objects within the same frame and can also distinguish one from another by using their coordinates. The coordinates of the current objects are compared to the previous mask obtained, therefore making it possible to recognize if an object of interest has moved.
9. *Counting:* This function is able to number, count and display matches in the current frame and throughout the video. This is established by using the object’s coordinates within the frame to differentiate multiples objects and to count the number of consecutive frames in which those objects have appeared. If this number increases, it means the object is still in the frame and this is counted as a match. If the object leaves the scene for a specified number of frames after being identified as a match, it is included in the total count.
10. *Last frame Loop:* In this last step the algorithm then checks if the current frame is the last. If not the algorithm restarts at Step 3; otherwise the process ends.

## 3. Validation Test

### 3.1. Focal Species: Koala

The koala (*Phascolarctos cinerus*) was chosen as the focal species for several reasons: it is an iconic native marsupial species of Australia whose status is listed as vulnerable in parts of the region [31] and its sedentary nature makes it ideal to trial the system described in this paper. Northern and eastern koala populations have declined substantially over recent years, mainly due to factors including loss of habitat, disease, road kill and predation by dogs [31]. A Senate inquiry into the status and health of the koala population in Australia was commissioned in 2010 [32]. One of the outcomes from the inquiry recommended a “national monitoring, evaluation and population estimation program for koalas” [31].

### 3.2. Study Area

The site selected for this experiment was located on the Sunshine Coast, 57 km north of Brisbane, Queensland, Australia. This site is a large rehabilitation enclosure where the number of koalas is determined in advance, which enables the accuracy of our counting algorithms to be assessed (ground-truthing).

**Figure 12:**
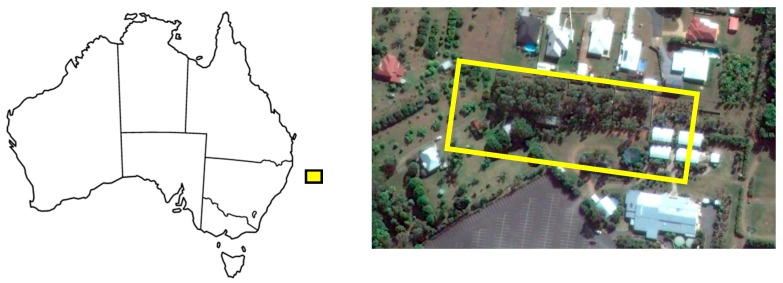
Location map of the study area, which is on the Sunshine Coast, Queensland, Australia.

The elevation of the treetop canopy varies between 20 m and 30 m, and is more densely populated in the western side than the eastern side (Figure 12). The experiment was conducted on 7th November 2014, with flights taking place between 7:10 a.m. and 8 a.m. Flying early in the morning provides optimal temperature difference for the target species (koalas), which makes it easier to distinguish between koalas, vegetation and soil. On that particular date, and during the specified time window, air temperatures oscillated between 21 and 24 °C. The flight could not start earlier due to fog and light rain, which actually contributed to lower soil and vegetation temperatures than would usually be the case for that time of day. During the flight window koala temperatures remained between 27 and 32 °C, while soil and vegetation remained below 25°.

### 3.3. Data Acquisition

**Figure 13:**
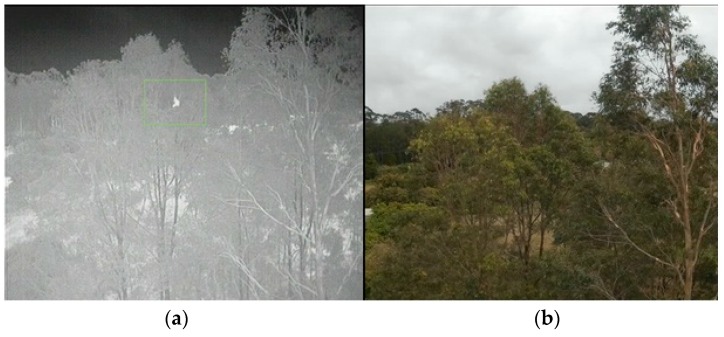
(**a**) Thermal image (**b**) digital lateral view at 20 m.

**Figure 14:**
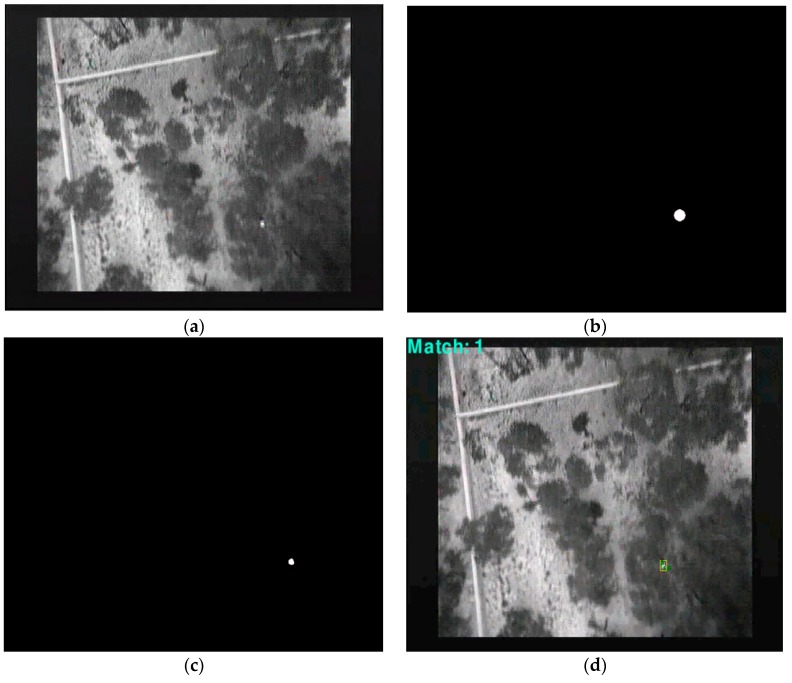
(**a**) Image of Canopy with Detection at 60 m (**b**) Mask created (**c**) Background removal, (**d**) Tracking and displaying.

**Figure 15:**
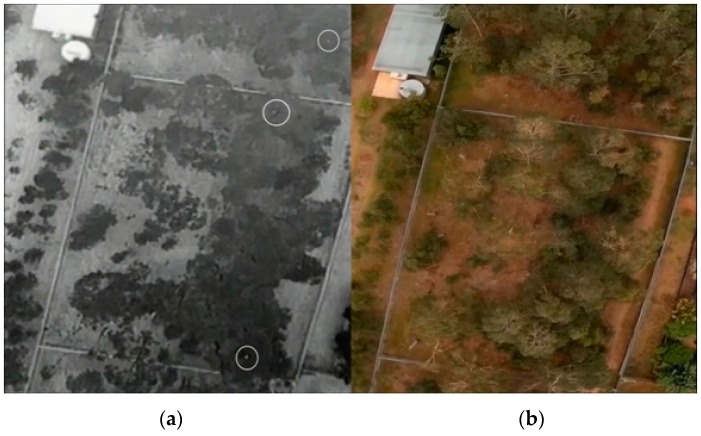
(**a**) Thermal image (**b**) digital RB6 capture at 80 m.

Both RGB video and thermal video were obtained simultaneously during the flights over the study area. Survey flights were performed at 60 m and 80 m in autonomous mode following a “lawn mowing” pattern (e.g., Figure 13 and Figure 14), with ground speeds of 2.5 m/s and 4 m/s, respectively. In these flights the gimbal was set to maintain the cameras in down-looking position. Additionally a flight was performed in manual mode at around 20 m flight height, keeping a regular ground speed and with the cameras in lateral-looking position (Figure 15). Camera specifications for both thermal and RGB are given in Table 1.

**Table 1:** Camera specifications for RGB and FLIR Thermal camera.

	Mobius RGB Camera	FLIR Thermal Camera
Size	61 mm × 35 mm × 18 mm	44.5 mm × 44.5 mm × 55 mm
Weight	38 g	72 g
Spectrum Wavelength	Visible RGB	7.5 -13.5 µm
Resolution	1080 p	640 × 510
Focal Length	2.1 mm	25 mm
Frame Rate	30 fps	9 fps

## 4. Results and Discussion

**Figure 16:**
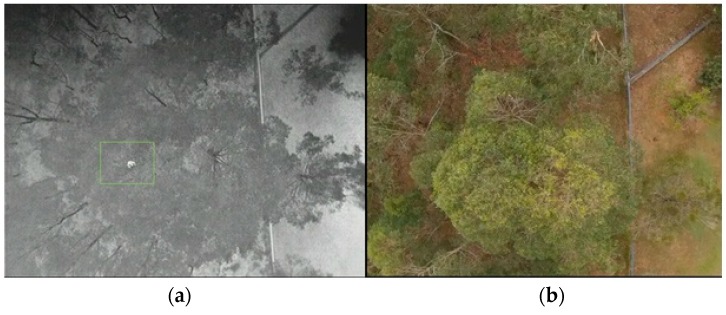
(**a**) Thermal image (**b**) digital R6B capture at 60 m.

The detection algorithms described in Section 2.2.1 and Section 2.2.2 were applied post-flight on the recorded imagery. The test flights successfully located koalas of different shapes and sizes. Figure 16a,b are examples of results at a height of 60 m. Figure 16a is a greyscale image with thermal imagery tracking. Figure 16b is a digital image of the same view illustrating the difficulty of visually spotting the koala. Similarly, Figure 13a,b show a greyscale thermal image of a koala identified alongside the real world image from a lateral perspective. 

Figure 15 shows an example of an image from footage obtained at 80 m where a thermal signature (e.g., koala) has been detected while searching above the canopy. We found that at this distance, and at heights greater than 60 m, confirming the detection of a particular thermal signature is difficult but this is also highly dependent on the camera characteristics and settings as well as the thermal differential between the target and other objects. Moreover, we found it challenging to distinguish between thermal signatures of different species, due to the lower resolution and difference in target object size relative to the image size. Consequently flying at such heights can feasibly result in false positives. The TMBM algorithm was tested on footage taken while flying at a height lower than 60 m.

The steps of TMBM algorithm were applied as follows. For Step 1, we defined a reference template to search for matching templates within the video. In this case the template was based on a sample of images of koalas seen from above at given altitudes. Processing was then applied to the template to improve the contrast between the shape and the background, as per Step 2. Step 3 involved execution of a search for the templates in the video. Due to the absence of local features to describe the koalas at different altitudes (20 m, 30 m, 60 m and 80 m), it was not possible to apply other types of approaches such as key point descriptors, parameterized shapes or colour matching.

Following Steps 4 and 5, the coordinates of the possible matches were saved as a vector for a follow-up check and a circle or a square box was drawn with the centre of the coordinates creating an image mask (Figure 14b). A logic AND operation was applied to remove what was considered as background and to focus on the region of interest (Figure 14c). Tracking was carried out as described in Step 6 of the TMBM algorithm. This was achieved by assuming the following:

1. If the target wildlife (e.g., koala) is found in at least 10 consecutive frames it is counted as a match
2. The target wildlife (e.g., koala) that has been identified cannot make big jumps in location (coordinates) between consecutive frames.
3. A horizontal displacement of the target wildlife (e.g., koala) is expected to be within the circle surrounding the target in the mask
4. The size of the target wildlife (e.g., koala) cannot suddenly increase drastically (area in pixels).
5. If the target wildlife (e.g., koala) being tracked is not found for 10 consecutive frames it is considered lost or out of the frame.

**Figure 17:**
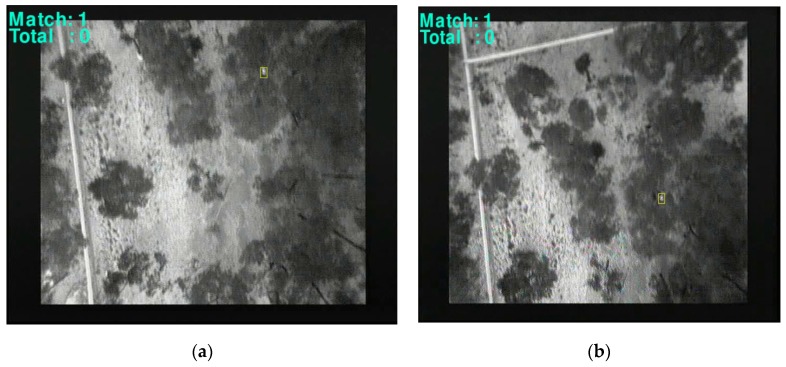
Koala tracking and detection above the canopy at 60 m.

**Figure 18:**
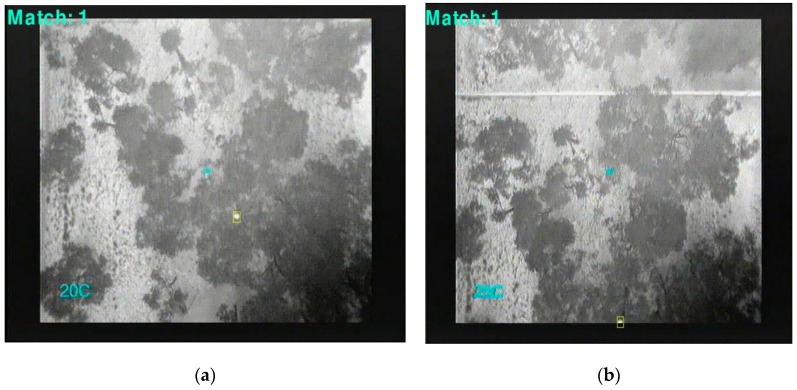
Koala tracking and detection above the canopy at 30 m.

**Figure 19:**
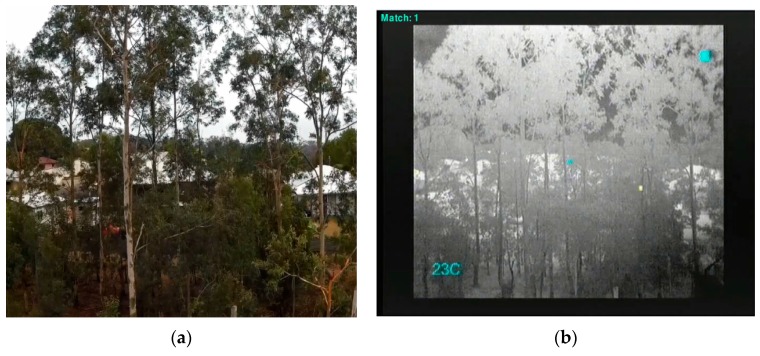
(**a**) Digital image (**b**) koala tracking and detection above the canopy with image of canopy with side – lateral view detection at 20 m.

Figure 14d displays a rectangle containing the identified object of interest based on the above procedure. Results of detection, tracking and counting over multiple frames and at different altitudes (60 m, 30 m and 20 m) are shown in Figure 17, Figure 18 and Figure 19 respectively.

**Figure 20:**
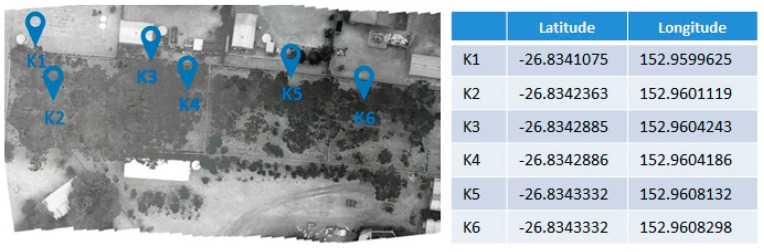
Orthomosaic thermal image with GPS location of detected koalas.

In order to evaluate the accuracy of detection, the GPS locations of the detected koalas (Figure 20) were compared with counts obtained by people on the ground. Even though in some cases a single koala was difficult to locate, the vertical traveling of the UAV above the canopy gave the camera a new angle of vision, locating the koala in almost all cases. We filtered all the false positives as described in Section 3, Steps 1 to 5, giving a clean and accurate result. This comparison can be seen in Table 2. Average detection times were defined as the time since the koala was first detected until the algorithm was able to mark it as a “real” match. These were recorded by the algorithm to determine the optimal altitude of detection. As the sampling frequency of the thermal camera is 9 fps, all detections were made in over one second changing as the altitude increases.

**Table 2:** Comparison of number of detections, actual number of koalas, average detection time and average false positives.

Altitude	Number of Detections	Actual Number of Koalas	Average Detection Time (s)	Average False Positives
20 m	6	6	1.3	0
30 m	6	6	1.6	0
60 m	5 to 6	6	2.1	1.5

At altitudes of 20 to 30 m the average detection time was 1.5 s and the false positives were completely filtered by the algorithm. At altitudes above 30 m the algorithm took more time to detect the koala since the UAV had to fly over and then past the koala to detect it. This fractionally increased the number of false positives, although these only appeared in the detection task and was removed in the tracking task. 

## 5. Conclusions

UAVs have proven to be effective at carrying out high-resolution and low-disturbance wildlife aerial surveys in a convenient and timely fashion, especially in habitats that are challenging to access or navigate at ground level [9]. However detecting or counting the species of interest from the large volumes of imagery collected during the flights has often proven to be very time consuming [9]. Other important issues are UAV regulations, operational costs, public perception [9,17], despite increasing effort in this area [15,16]. 

This paper addresses the challenge of automated wildlife detection in UAV imagery by describing a system that combines UAVs with thermal imaging capabilities and artificial intelligence image processing to locate wildlife in their natural habitats. The TMBM algorithm was able to detect the target koalas at the various locations and from various altitudes and produced an orthomosaic thermal image with GPS location of the species of interest (*i.e*., koala). The accuracy of detection at altitudes of 20 m, 30 m and 60 m was compared with ground truth detections, showing 100% accuracy at these altitudes. In cases where the species is difficult to locate, vertical traveling of the UAV above the canopy is able to give the camera a new angle of vision and hence improve detection. 

The system can be applied to including pest detection (e.g., wild dogs, wild cats, wild pigs, and dingos), locating protected or relocated animals or for search and rescue missions by adjusting the code and templates accordingly. The optimal detection height is likely to vary depending on various factors such as the size, thermal footprint, behaviour and habitat of the target species. This becomes more complex when there are several species of interest. For example, it may be desirable to detect not only the target species, but also its key predators and if applicable, its preferred prey. The GPS orthomosaic map displaying the locations of the desired animals may be used to assist developers and stakeholders to better understand the species’ population distribution and abundance before approval is granted for development, or before construction begins in their habitat. Improvements to template accuracy and detection are possible by improving the quality of the original templates. The mask can be updated to adjust to the area being overflown and to distinguish between different animals as well as different sizes or temperatures. Implementing a dynamic template would increase the accuracy of detecting koalas and mammals of different sizes. A dynamic template to account for different possible orientations, positions and angles would mean that the template could be changed and updated during the flight in real time with respect to the object of interest. Radio communication using short ranged antennas could be combined with the system and algorithms to match the thermal imagery with a tagged animal. A copy of the software, algorithms and a User Manual is also available. Please contact felipe.gonzalez@qut.edu.au for more information.

## References

[1] Cristescu R.H., Foley E., Markula A., Jackson G., Jones D., Frère C. (2015). Accuracy and efficiency of detection dogs: A powerful new tool for koala conservation and management. Sci. Rep..

[2] Burton A.C., Neilson E., Moreira D., Ladle A., Steenweg R., Fisher J.T., Bayne E., Boutin S. (2015). Wildlife camera trapping: A review and recommendations for linking surveys to ecological processes. J. Appl. Ecol..

[3] Witmer G.W. (2005). Wildlife population monitoring: Some practical considerations. Wildl. Res..

[4] Christiansen P., Steen K.A., Jørgensen R.N., Karstoft H. (2014). Automated detection and recognition of wildlife using thermal cameras. Sensors.

[5] Gaston K.J., Fuller R.A. (2009). The sizes of species’ geographic ranges. J. Appl. Ecol..

[6] Murray J.V., Low Choy S., McAlpine C.A., Possingham H.P., Goldizen A.W. (2008). The importance of ecological scale for wildlife conservation in naturally fragmented environments: A case study of the brush-tailed rock-wallaby (*petrogale penicillata*). Biol. Conserv..

[7] Schaub M., Gimenez O., Sierro A., Arlettaz R. (2007). Use of integrated modeling to enhance estimates of population dynamics obtained from limited data. Conserv. Biol..

[8] Ditmer M.A., Vincent J.B., Werden L.K., Iaizzo P.A., Garshelis D.L., Fieberg J.R. (2015). Bears show a Physiological but Limited Behavioral Response to Unmanned Aerial Vehicles. Curr. Biol..

[9] Chabot D., Bird D.M. (2015). Wildlife research and management methods in the 21st century: Where do unmanned aircraft fit in?. J. Unmanned Veh. Syst..

[10] Anderson K., Gaston K.J. (2013). Lightweight unmanned aerial vehicles will revolutionize spatial ecology. Front. Ecol. Environ..

[11] Linchant J., Lisein J., Semeki J., Lejeune P., Vermeulen C. (2015). Are unmanned aircraft systems (UAS) the future of wildlife monitoring? A review of accomplishments and challenges. Mamm. Rev..

[12] Mulero-Pázmány M., Barasona J.Á., Acevedo P., Vicente J., Negro J.J. (2015). Unmanned aircraft systems complement biologging in spatial ecology studies. Ecol. Evol..

[13] Soriano P., Caballero F., Ollero A. RF-based particle filter localization for wildlife tracking by using an UAV. Proceedings of the 40th International Symposium on Robotics.

[14] Van Gemert J.C., Verschoor C.R., Mettes P., Epema K., Koh L.P., Wich S.A., Agapito L., Bronstein M.M., Rother C. (2015). Nature conservation drones for automatic localization and counting of animals. Computer Vision—ECCV 2014 Workshops, Part I.

[15] Williams B.P., Clothier R., Fulton N., Johnson S., Lin X., Cox K. Building the safety case for uas operations in support of natural disaster response. Proceedings of the 14th Aiaa Aviation Technology, Integration, and Operations Conference.

[16] Cork L., Clothier R., Gonzalez L.F., Walker R. (2007). The future of UAS: Standards, regulations, and operational experiences [workshop report]. IEEE Aerosp. Electron. Syst. Mag..

[17] Vincent J.B., Werden L.K., Ditmer M.A. (2015). Barriers to adding UAVs to the ecologist’s toolbox. Front. Ecol. Environ..

[18] Bevan E., Wibbels T., Najera B.M.Z., Martinez M.A.C., Martinez L.A.S., Martinez F.I., Cuevas J.M., Anderson T., Bonka A., Hernandez M.H. (2015). Unmanned aerial vehicles (UAVs) for monitoring sea turtles in near-shore waters. Mar. Turt. Newsl..

[19] Vermeulen C., Lejeune P., Lisein J., Sawadogo P., Bouché P. (2013). Unmanned aerial survey of elephants. PLoS ONE.

[20] Hodgson A., Kelly N., Peel D. (2013). Unmanned aerial vehicles (UAVs) for surveying marine fauna: A dugong case study. PLoS ONE.

[21] Chabot D., Bird D.M. (2012). Evaluation of an off-the-shelf unmanned aircraft system for surveying flocks of geese. Waterbirds.

[22] Dos Santos G.A.M., Barnes Z., Lo E., Ritoper B., Nishizaki L., Tejeda X., Ke A., Han L., Schurgers C., Lin A. (2014). Small unmanned aerial vehicle system for wildlife radio collar tracking. Proceedings of the 2014 IEEE 11th International Conference on Mobile Ad Hoc and Sensor Systems (MASS).

[23] Mulero-Pázmány M., Stolper R., van Essen L.D., Negro J.J., Sassen T. (2014). Remotely piloted aircraft systems as a rhinoceros anti-poaching tool in Africa. PLoS ONE.

[24] Jones G.P.I.V., Pearlstine L.G., Percival H.F. (2006). An assessment of small unmanned aerial vehicles for wildlife research. Wildl. Soc. Bull..

[25] Korner F., Speck R., Goktogan A., Sukkarieh S. Autonomous airborne wildlife tracking using radio signal strength. Proceedings of the 2010 IEEE/RSJ International Conference on Intelligent Robots and Systems (IROS).

[26] Leonardo M., Jensen A., Coopmans C., McKee M., Chen Y. A Miniature Wildlife Tracking UAV Payload System Using Acoustic Biotelemetry. Proceedings of the 2013 ASME/IEEE International Conference on Mechatronic and Embedded Systems and Applications Portland.

[27] DJI. (n.d.) S800 EVO.

[28] FLIR. (n.d.) FLIR.

[29] IPI Learning Kangaroos Boxing in Infrared [Video file]. https://www.youtube.com/watch?v=aBsvoWfHWXQ.

[30] Trail cameras Imagers for watching wildlife [Video file]. https://www.youtube.com/watch?v=ZpBgt91Qor8.

[31] Shumway N., Lunney D., Seabrook L., McAlpine C. (2015). Saving our national icon: An ecological analysis of the 2011 Australian Senate inquiry into the status of the koala. Environ. Sci. Policy.

[32] Senate Environment and Communications References Committee Completed inquiries 2010-2013: The Koala-Saving Our National Icon. http://www.aph.gov.au/Parliamentary_Business/Committees/Senate/Environment_and_Communications/Completed%20inquiries/2010-13/koalas/index.

